# Construction and Application of EGCG-Loaded Lysozyme/Pectin Nanoparticles for Enhancing the Resistance of Nematodes to Heat and Oxidation Stresses

**DOI:** 10.3390/foods10051127

**Published:** 2021-05-19

**Authors:** Yu Zhang, Liufeng Lin, Hao Cui, Bin Li, Jing Tian

**Affiliations:** 1 College of Food Science and Technology, Huazhong Agricultural University, Wuhan 430070, China; zhang.y@webmail.hzau.edu.cn (Y.Z.); 635383426@qq.com (L.L.); vqh825@alumni.ku.dk (H.C.); libinfood@mail.hzau.edu.cn (B.L.); 2 Key Laboratory of Environment Correlative Dietology, Huazhong Agricultural University, Ministry of Education, Wuhan 430070, China; 3 Functional Food Engineering & Technology Research Center of Hubei Province, Wuhan 430070, China

**Keywords:** EGCG, lysozyme, pectin, nanoparticles, *Caenorhabditis elegans*, stress defense

## Abstract

Novel nanoparticles (NPs) were constructed with lysozyme (LY) and pectin (Ps) through self-assembly, which were used as a carrier to encapsulate epigallocatechin-3-gallate (EGCG). The binding of EGCG and LY is a static quenching process. Hydrogen bonds might play a major role in the formation of NPs, which has also been verified by a lower binding constant of EGCG with LY/Ps NPs. Meanwhile, EGCG could lead to conformational and microenvironmental changes of LY, resulting in more folding of LY secondary structures. In addition, attaching Ps to LY might inhibit LY aggregation induced by addition of free EGCG. At the LY/Ps mass ratio of 1:1, the constructed LY/Ps NPs had a high EGCG-loading capacity without a significant change in mean particle size, thus, our NPs could be used as an effective nanocarrier for loading EGCG. In vivo, compared with free EGCG, EGCG loaded onto LY/Ps NPs significantly increased *Caenorhabditis elegans*’ (*C. elegans*) resistance to heat stress and oxidative injury and prolonged their lifespan. This study provides theoretical basis and reference for constructing nanoactive substance carriers so as to improve the resistance of organisms to heat stress and oxidative damage and to increase their survival rate and extend their lifespan under environment stresses.

## 1. Introduction

**Figure 1:**
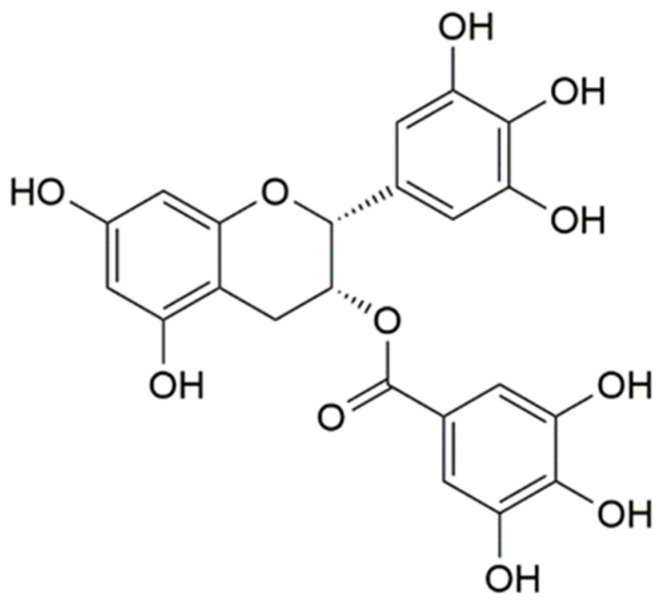
Structure of epigallocatechin-3-gallate.

Epigallocatechin-3-gallate (EGCG, Figure 1) is the most important component of catechol, which is well known for its natural antioxidant activity [1]. Many epidemiological and preclinical trials have shown that EGCG can reduce the risk of cancer, cardiovascular, and neurodegenerative diseases [2]. Due to its strong antioxidant and pharmacological activities, EGCG can be used as an antioxidant and synergist in food and pharmaceuticals [3]. However, EGCG is unstable and prone to autoxidation and oxidation into less active substances; it has low bioavailability when taken directly [4]. Moreover, EGCG is easily soluble in high polar solvents, such as water and ethanol, which has poor liposolubility and membrane permeability, and its high polarity reduces its cellular adsorption capacity [5,6]. Therefore, the EGCG encapsulation inside drug delivery systems is one of the strategies currently used in overcoming these drawbacks. Nanoparticles (NPs) are often used as drug delivery vehicles due to their small size, good biocompatibility, and low toxicity [7]. To promote the application of EGCG in food and pharmaceuticals, loading EGCG into nanocarriers can improve its performance, such as stability, prevention from oxidation, and bioavailability [8,9,10].

In recent years, nutrition and drug delivery carriers based on self-assembly of food biomolecules have become a hot research topic [11,12,13]. Protein/polysaccharide-based carriers have many advantages, such as non-toxicity, biocompatibility, biodegradability, and so on [14,15]. Lysozyme (LY) is a natural antibacterial protein widely found in plant sap, animal secretions, tears, saliva, milk, poultry eggs, and some bacteria. LY not only has cell wall enzyme activity and can hydrolyze β-1,4 glycosidic bonds between N-acetyl cell wall acid and N-acetylglucosamine in peptidoglycan, but also has antibacterial peptide activity, which is highly related to the structure, charge distribution, and surface hydrophobicity of LY [16]. LY is used as a natural and safe preservative for food preservation and is widely used in the pharmaceutical industry as a non-specific immune factor with pharmacological efficacy [17]. With small molecular weight, LY can form tightly-structured complexes, together with polysaccharide, and its isoelectric point is alkaline, thus, it can form a complex with polysaccharide, with pH ranging from neutral to weak alkaline [18]. Pectin (Ps) is one of the most widely used natural polysaccharide macromolecular compounds present in fruits and vegetables and it is also a hydrophilic gum. Ps is widely used in the food and medicine industry because of its natural sources and high nutrition [19]. Ps can be used as a thickener, emulsifier, stabilizer, and gelling agent in food production [20] and it can also be used as dietary fiber in health food and medicine. In addition, Ps has good biocompatibility and low toxicity [21], high tissue adhesion, high colonic localization, and decomposition abilities, and thus it can be used as a delivery carrier for colonic localization and drug release in the pharmaceutical field [22]. The larger the molecular weight, the stronger the interaction between polysaccharide and protein, which promotes the formation of insoluble complexes. Therefore, low molecular weight pectin tends to be selected to study the interaction between Ps and protein [23,24]. Based on the above-mentioned properties of pectin, protein-polysaccharide soluble complexes with different structures and characteristics are prepared to broaden the application of Ps in the field of food and medicine.

In this paper, lysozyme and pectin were used as carriers to prepare green and natural NPs for loading bioactive substance EGCG by one-step self-assembly. The conformational changes of LY were characterized by fluorescence quenching, synchronous fluorescence, and circular dichroism (CD). The interaction, binding constant, and binding force between LY and EGCG were investigated. Dynamic light scattering (DLS) and high performance liquid chromatography (HPLC) methods were used to determine the particle size and the entrapment efficiency of EGCG. In this study, the enhancement of resistance to heat and oxidative stress caused by the encapsulation of EGCG with LY/Ps nanoparticles in an in-vivo model of *Caenorhabditis elegans* (*C. elegans*) was demonstrated for the first time, to our knowledge. This may provide insights for the application and the biological activity evaluation of phytochemicals.

## 2. Materials and Methods

### 2.1. Materials

The N2 wild-type *Caenorhabditis elegans* (*C. elegans*) strain and *E. coli* OP50 were purchased from Caenorhabditis genetics Center (University of Minnesota, Minneapolis, MN, USA). Lysozyme (enzyme activity > 20 ku/mg) was obtained from VWR International, Inc. (St. Louis, MO, USA). Pectin (galacturonic acid (dry basis) ≥ 74.0%) was purchased from Sinopharm Chemical Reagent Co., Ltd. (Shanghai, China). Epigallocatechin-3-gallate (EGCG) (98% purity) was provided from Chengdu purefa Technology Development Co., Ltd. (Chengdu, China).

### 2.2. Preparation Conditions of Lysozyme/Pectin Nanoparticles (LY/Ps NPs)

The nanoparticles were prepared as previously reported [25]. Briefly, a certain mass of LY and Ps samples were accurately weighed and dissolved in 10 mM of PBS (pH 7.0), respectively, stirred at room temperature, and then hydrated in the refrigerator overnight. The LY/Ps NPs were obtained by mixing LY and Ps stock solution at the volume ratio of 1:1 to obtain LY/Ps NPs with the mass ratio of LY to Ps (LY:Ps) of 1:1 or 1:2. The final concentration of LY was 0.5 mg/mL, and the final concentration of Ps was 0.5 mg/mL or 1 mg/mL.

### 2.3. Preparation of EGCG-Loaded Lysozyme/Pectin Nanoparticles (EGCG-LY/Ps NPs)

An EGCG sample was weighed and dissolved in 10 mM of acetate buffer (pH 4.5). EGCG solution was added to the LY/Ps NPs at a volume ratio of 1:9 and different final concentrations of EGCG were obtained according to different molar ratios of EGCG to LY (0:1, 1:1, 2:1, 4:1, 8:1, 16:1, 32:1, 64:1, and 128:1).

### 2.4. Determination of Interaction of LY/Ps NPs with EGCG by Fluorescence Spectroscopy

The sample preparation method was the same as described in Section 2.3, and the molar mass ratio of EGCG to LY was 0:1, 0.2:1, 0.4:1, 0.8:1, 1:1, 2:1, 3:1, and 4:1. Fluorescence quenching spectra of LY in EGCG-LY/Ps NPs were measured using a HITACHI F4600 fluorescence spectrophotometer. The excitation wavelength (λ_ex_) was fixed at 280 nm. The excitation and emission slit widths were set as 2.5 nm and 5 nm, respectively. The scanning range was set as 300~500 nm. Finally, the binding constants, binding sites, and thermodynamic parameters of EGCG and LY and EGCG and LY/Ps NPs were calculated according to the corresponding equation, respectively. Synchronous fluorescence measurement parameters were set as follows. The wavelength difference Δλ was set as 15 nm and 60 nm, and the synchronous fluorescence spectra were recorded from 265 to 360 nm and 220 to 360 nm, respectively.

The dynamic quenching was a process of interaction between EGCG and LY, which was determined in accordance with the Stern–Volmer equation [26]:

F_0_/F = 1 + K_q_ τ_0_ [EGCG] = 1 + K_sv_ [EGCG] (1)

where F_0_ is the fluorescence intensity of LY without EGCG; F is the fluorescence intensity of LY with additional different concentrations of EGCG; [EGCG] is the concentration of EGCG; K_sv_ is the Stern–Volmer dynamic quenching constant; K_q_ is the bimolecular rate quenching constant; and τ_0_ is the average lifetime of biomacromolecule without the quencher, which is set as 10^−8^ s.

### 2.5. Calculation of Binding Constants and Thermodynamic Parameters

When the temperature change was small, the enthalpy value (ΔH) could be viewed as a constant. The entropy change (ΔS) and Gibbs free energy (ΔG) were calculated to determine the interaction force type. The thermodynamic parameters were calculated according to the following formula [27]:

ΔG = −RT lnK_a_ (2)

lnK_2_/K_1_ = (1/T_1_ − 1/T_2_) ΔH/R (3)

ΔG = ΔH − TΔS(4)

where R is the gas constant; K is the binding constant related to temperature; and K_1_ and K_2_ are the binding constants at the temperature T_1_ and T_2_, respectively_._

### 2.6. Determination of Interaction between LY/Ps NPs and EGCG by Circular Dichroism Chromatography

The sample was prepared as described in Section 2.3, with the mass ratio of LY to Ps of 1:1 and the molar mass ratios of EGCG to LY of 0:1, 1:1, 2:1, and 4:1. The final concentration of LY was 0.1 mg/mL by dilution with PBS. Measurement parameters were set as follows: scanning speed, 50 nm/min; slit width, 1 nm; response time, 1 s; 3 scans for each sample; resolution, 0.1 nm; and quartz cuvette, 0.1 cm. Circular dichroism (CD) profiles within the range of 190–250 nm were recorded before and after the interaction between LY and EGCG. The percentages of protein secondary structures (α-helix, β-sheet, β-turn, and random coil) were calculated by Yang’ Reference fitting.

### 2.7. Determination of EGCG-LY/Ps NPs Particle Size Distribution

The particle size distribution, polydispersity index (PDI), and ξ-potential of EGCG-LY/Ps NPs were analyzed using a nanoparticle size distribution analyzer (Zetasizer Nano, Malvern Instruments Ltd., Malvern, Worcestershire, UK). All tests were performed at room temperature, with the scattering angle set as 173°. Three replicate experiments were performed for each sample.

### 2.8. Determination of EGCG-LY/Ps NPs Loading Rate

EGCG-LY/Ps NPs (LY:Ps = 1:1) were loaded into ultrafiltration centrifuge tubes (MW:3K) and centrifuged at 4000 rpm for 30 min. After moderate dilution of the filtrate, the content of EGCG was detected by waters 2695 high performance liquid chromatography (HPLC) under the following detection conditions: C18 reversed-phase column (5 μm, 39 × 150 mm, Atlantis, Waters, Milford, MA, USA); column temperature, 25 °C; injection volume, 10 μL; flow rate, 1 mL/min; detection wavelength, 280 nm. The EGCG encapsulation rate (EE) and its loading capacity (LC) by NPs were calculated as follows [28]:

EE = (T − T_0_)/T × 100% (5)

LC = (T − T_0_)/T_w_ × 100% (6)

where T is the total amount of EGCG (mg/mL); T_0_ is the amount of free EGCG (mg/mL); and T_w_ is the total weight of NPs (mg/mL).

### 2.9. Heat Stress Assay

After 5 days of incubation under different dosing conditions, 100 nematodes were selected and put into 96-well plates, with each well containing 100 μL of S-basal buffer and subjected to heat stress in an incubator at 37 °C. The number of nematode deaths per hour was recorded until all the nematodes died. There were three replicates for each group, with 100 nematodes in each replicate.

### 2.10. Oxidative Stress Assay

After being synchronized for 64 h, the *C. elegans* were transferred to the incubation plates under different drug administration conditions. The nematodes were divided into the control group, EGCG group, and EGCG-LY/Ps NPs group, according to drug administration conditions. After 5 days of administration, nematodes were put into 96-well plates (each well contained 100 μL of paraquat solution at a concentration of 50 mM), and nematode mortality was recorded hourly until all the nematodes died. Nematode mortality was judged by gently touching the nematode body with a picker. If it did not respond to this mechanical stimulus and its pharynx stopped moving, it was viewed as dead. The experiments were performed with three biological replicates for each group. There were 100 nematodes for each replicate.

### 2.11. Statistical Analysis

The data were expressed as mean ± SD, and the mean value of at least three replicates for each experiment were calculated. Statistical analysis was performed using SPSS 18.0, and survival curves were plotted by GraphPad Prism 7.04. The difference in survival rate between groups was determined by using the Chi-test. *p* < 0.05 was considered statistically significant.

## 3. Results and Discussion

### 3.1. Interaction of EGCG with Lysozyme and Lysozyme/Pectin Nanoparticles (LY/Ps NPs)

**Figure 2:**
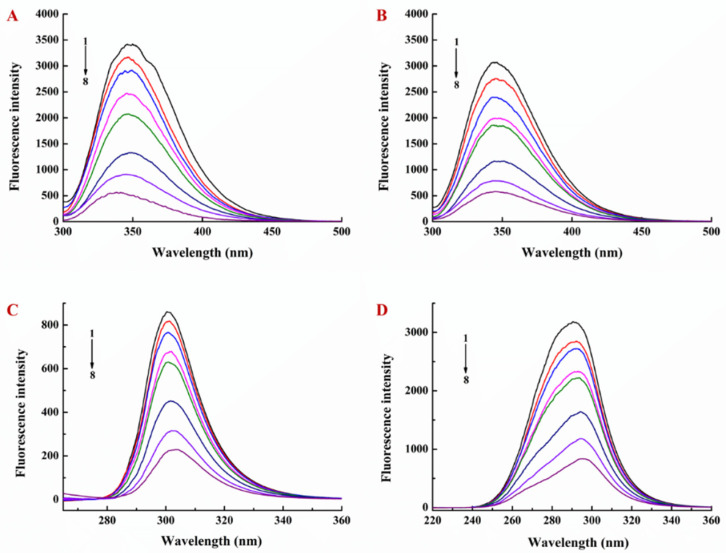
Fluorescence emission spectra of EGCG-lysozyme (**A**) at 25 °C and (**B**) at 35 °C. Synchronous fluorescence spectra of EGCG-lysozyme at 25 °C, (**C**) Δλ = 15 nm, and (**D**) Δλ = 60 nm.

Firstly, the interactions between different concentrations of EGCG and LY, as well as those contained in NPs, were investigated by fluorescence spectroscopy. LY has been previously reported to have a strong fluorescence peak near 346 nm [29], based on which, in this study, we investigated the fluorescence emission spectra within the range of 300–500 nm at a fixed concentration of LY (0.5 mg/mL) and different molar ratios of EGCG to LY (0:1, 0.2:1, 0.4:1, 0.8:1, 1:1, 2:1, 3:1, and 4:1). As shown in Figure 2A,B, with the increasing EGCG concentration, the fluorescence intensity of the LY emission peak decreased, indicating that the interaction between EGCG and LY caused the regular quenching of LY endogenous fluorescence. With the increasing temperature, the intensity of LY endogenous fluorescence also decreased [27].

To further investigate the effect of EGCG on LY fluorescence quenching, fluorescence intensity was measured at 25 °C and 35 °C. The maximum diffusive collision quenching rate constant of the quenching agent for biological macromolecules was reported to be 2.0 × 10^10^ L/mol·s in the previous study [30]. The dynamic quenching constant (K_sv)_, reflecting the interaction of EGCG with LY, was calculated as 3.13 × 10^4^ L·mol^−1^ at 25 °C and 2.94 × 10^4^ L·mol^−1^ at 35 °C. These quenching rates (K_q_) of LY fluorescence by EGCG were greater than 2.0 × 10^10^ L/mol·s (above-mentioned critical value), indicating that the interaction of EGCG with LY was a static quenching, which was confirmed by another observation that, with the increasing temperature, the stability of the NPs was decreased and accordingly K_sv_ was decreased, indicating that the quenching of LY by EGCG was a static quenching.

**Figure 3:**
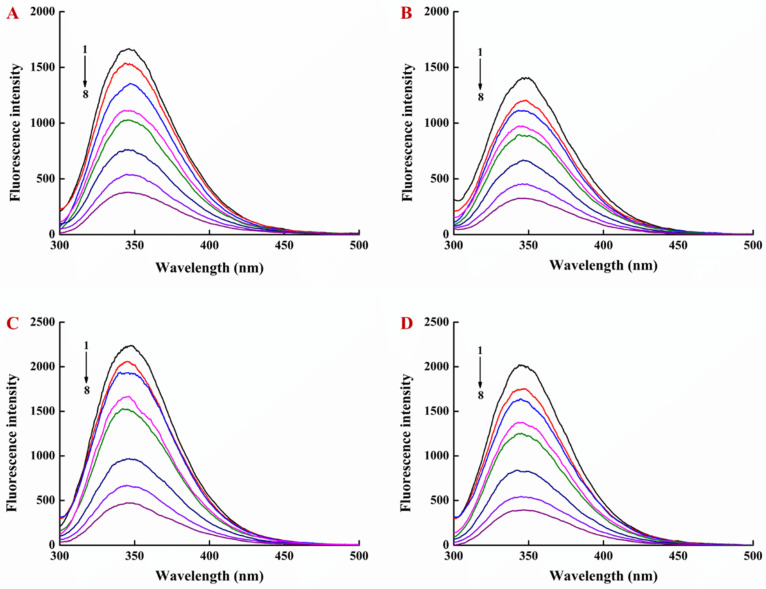
Fluorescence emission spectra of EGCG-lysozyme/pectin nanoparticles with lysozyme/pectin mass ratio of 1:1 (**A**) at 25 °C and (**B**) at 35 °C and with lysozyme/pectin mass ratio of 1:2 (**C**) at 25 °C and (**D**) at 35 °C.

Figure 3 showed the fluorescence quenching spectra of LY in LY/Ps NPs by EGCG. The fluorescence intensity of the LY emission peak was gradually decreased with the increasing EGCG concentration, and the intensity of LY endogenous fluorescence was also gradually decreased with the increasing temperature. Compared to that, in the LY solution, the fluorescence intensity of LY in LY/Ps NPs was reduced and the fluorescence intensity of LY at a mass ratio of LY to Ps of 1:1 was lower than that at a mass ratio of 1:2, indicating that LY and Ps were more tightly bound through electrostatic interactions at the mass ratio of 1:1 than at the mass ratio of 1:2. In addition, at the LY/Ps mass ratio of 1:1, the dynamic quenching constant (K_sv_) was 2.27 × 10^4^ L·mol^−1^ at 25 °C and 2.15 × 10^4^ L·mol^−1^ at 35 °C, respectively. At the LY/Ps mass ratio of 1:2, K_sv_ was 2.77 × 10^4^ L·mol^−1^ at 25 °C and 2.61 × 10^4^ L·mol^−1^ at 35 °C, respectively. All the K_q_ values calculated based on the above K_sv_ values were greater than 2.0 × 10^10^ L/mol·s (constant K_q_), and these K_sv_ values were decreased with the increasing temperature, jointly indicating that the quenching of LY in LY/Ps NPs by EGCG was a static quenching.

**Figure 4:**
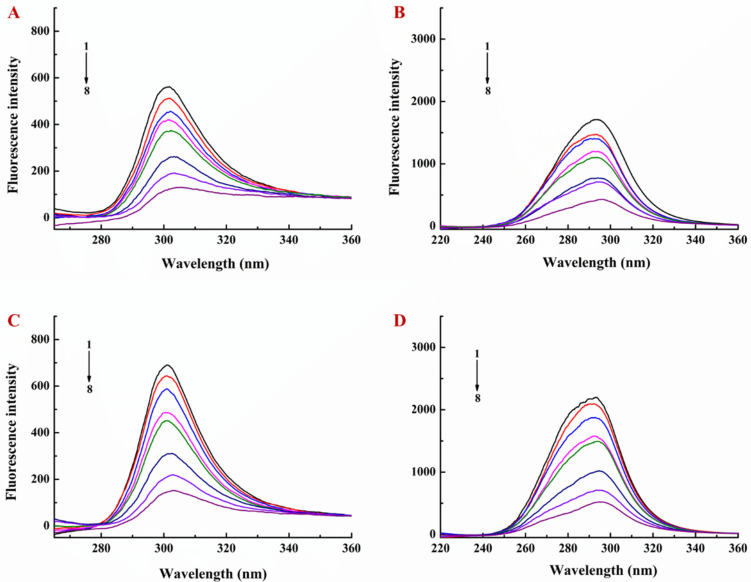
Effect of EGCG on synchronous fluorescence spectra of lysozyme/pectin nanoparticles at 25 °C with lysozyme/pectin mass ratio of 1:1, (**A**) Δλ = 15 nm and (**B**) Δλ = 60 nm, and with lysozyme/pectin mass ratio of 1:2, (**C**) Δλ = 15 nm and (**D**) Δλ = 60 nm.

Synchronized fluorescence spectroscopy can provide the information of polarity changes around the fluorescent amino acid residues, and emission wavelength changes can be used to determine protein conformation changes [31]. When the wavelength interval (Δλ) was fixed at 15 nm and 60 nm, the fluorescence emission peaks were the characteristic absorption peaks of tyrosine residues and tryptophan residues, respectively. The red shift shows that the hydrophobicity of the environment is decreased and the degree of peptide chain extension is increased, whereas the blue shift shows that the hydrophobicity of the environment is enhanced and the protein tends to fold [31,32]. In this study, the fluorescence intensity of both tyrosine and tryptophan residues was decreased, but the fluorescence intensity of tryptophan residues was more significantly decreased (Figure 2C,D). The fluorescence peak of tyrosine residues exhibited a slight red shift, and the fluorescence peak of tryptophan residues exhibited a strong red shift, indicating that the addition of EGCG increased the polarity and decreased the hydrophobicity of the residue microenvironment (Figure 2C,D). Similar results were observed after fixing the concentration of lysozyme and adding different amounts of EGCG to the LY/Ps NPs (Figure 4).

### 3.2. Binding Sites, Binding Constants, and Action Forces of EGCG Interaction with LY and NPs

The relationship between EGCG and LY is static quenching. The formula of fluorescence quenching intensity and quenching concentration is as follows [33]:

log [(F_0_ − F)/F] = log K_a_ + n log [EGCG] (7)

where F_0_ is the fluorescence intensity of LY without EGCG; F is the fluorescence intensity at additional different concentrations of EGCG; [EGCG] is the concentration of EGCG; K_a_ is the binding constant; and n is the number of binding sites.

In this study, the binding constants of EGCG and LY ranged within 10^4^–10^6^ L·mol^−1^ (Table 1), indicating that the formed complex between them was stable, which in turn indicated that the NPs formed by LY and Ps can be used as loading carriers for EGCG. Meanwhile, the binding constants and binding site number of LY with EGCG in LY/Ps NPs were reduced relative to the interaction of EGCG with pure LY solution. A possible explanation might lie in the fact that the negative charge of Ps and the positive charge of LY formed a complex through electrostatic interaction, which made it difficult for EGCG to approach LY, resulting in the decrease in binding site number and binding constant [25]. With the increasing Ps concentration, the negative charge density increased and the LY molecules with relatively neutral charge were more likely to bind to EGCG (Table 1). Temperature affected the diffusion coefficient and EGCG-LY complex stability. With the increase in temperature, the diffusion coefficient was increased and EGCG was separated from LY. With the temperature increasing from 25 °C to 35 °C, the binding constant of EGCG and LY/Ps complex decreased slightly, and such a decrease was less significant than that of EGCG and pure LY. The attachment of pectin to the surface of LY prevented EGCG from dissociating from LY and improved the stability of the EGCG-LY complex.

The interaction between polyphenols and proteins is mainly through electrostatic force, hydrogen bonding force, van der Waals force, and hydrophobic force [34]. The thermodynamic parameters of the interaction between polyphenols and LY can be used to determine the forces playing a role in the binding process. The relation between the above forces and thermodynamic parameters of tea polyphenol-biomolecule reaction is described as follows: hydrophobic forces make ΔH > 0 and ΔS > 0; hydrogen bonding and van der Waals forces make ΔH < 0 and ΔS < 0; electrostatic forces make ΔH ≈ 0 and ΔS > 0 [35]. The ΔG, ΔH, and ΔS of the binding of EGCG with LY alone or with LY in LY/Ps NPs were shown in Table 1. When the ΔG was negative, the binding process was spontaneous; when ΔH < 0, the binding was an exothermic reaction, thus a temperature increase was not conducive to the binding of EGCG with LY; when ΔS < 0, the forces might be hydrogen bond action forces and/or van der Waals forces [36]. Our data indicated that, when ΔH and ΔS were both negative, the forces between EGCG and LY were hydrogen bond action forces. The interaction with lysozyme is largely due to the presence of eight hydroxyl groups, which may form hydrogen bonds with the H-receptor of LY [37]. From the decreasing trend of the ΔH absolute value, it could be concluded that there were more hydrogen bonds in the interaction between EGCG and pure LY in solution than in the interaction between EGCG and the LY in LY/Ps NPs (Table 1). The instability of LY solutions induced by EGCG was improved by adding Ps to the LY solution, suggesting that stable protein-based nanocomplexes capable of loading high levels of polyphenols could be prepared using the interaction of proteins with polysaccharides.

### 3.3. Effect of EGCG on Secondary Structure of Lysozyme

CD is a reliable method for analyzing the secondary structure of proteins and it can explain the protein conformational changes caused by ligands [38]. Hydrogen bonds play a main role in stabilizing the secondary structure of proteins [39]. In this study, the CD spectra in the far ultraviolet region were used to determine the LY conformational changes induced by additional EGCG at different concentrations.

**Figure 5:**
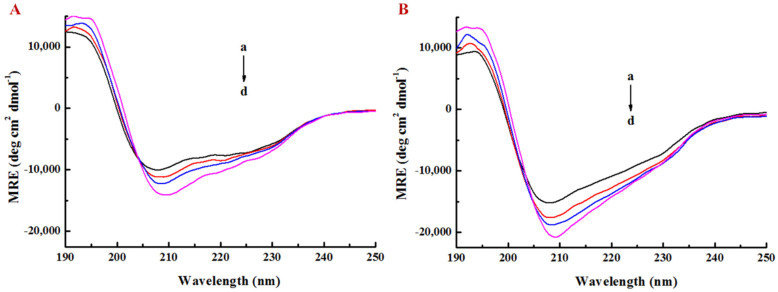
Far-UV CD spectra of (**A**) lysozyme and (**B**) lysozyme/pectin nanoparticles with lysozyme/pectin mass ratio of 1:1 in the presence and absence of EGCG at pH 7.0, [EGCG] = 0.1 mg/mL. The EGCG/lysozyme molar ratio was (a–d) 0:1, 1:1, 2:1, and 4:1.

LY molecule exhibited obvious negative valleys within the range of 205–235 nm, indicating that its secondary structure might contain an α-helix and β-sheet (Figure 5), since the typical α-helix has two negative valleys at about 208 nm and 222 nm and the β-sheet has one negative valley at 215 nm [40,41]. As shown in Figure 5A, the negative peaks at 208 nm and 222 nm were lowered continuously as the EGCG concentration increased. The secondary structure conformation of LY in PBS solution (pH 7.0) was fitted by using Young’s modulus to obtain the secondary structure composition with α-helix, β-sheet, β-turn, and random coil accounting for 21.0%, 46.7%, 3.0%, and 29.3%, respectively. When the molar ratio of EGCG to LY was 4:1, the α-helix content was increased from 21% to 34.5%, the β-sheet was decreased from 46.7% to 21.7%, and the β-turn was increased from 3% to 14.1% (Table S1). With the increasing EGCG concentrations, the negative peak at 208 nm was continuously lowered and the α-helix content of LY in LY/Ps NPs was gradually increased (Figure 5B and Table S1). The CD diagram of LY showed that the α-helix content was increased and the β-sheet content was decreased after the interaction between EGCG and LY, which might be attributed to the fact that the EGCG-LY interaction changed the direction of hydrogen bonds, leading to the conformational change of LY, thus resulting in the change in the folding state of the secondary structures, including the increase in the stability of the helix structure. The secondary structures of LY changed after adding Ps into PBS (pH 7.0), with the content of α-helix increased and the content of β-sheet decreased, indicating that electrostatic attraction might occur between the negative charge of Ps and the positive charge of LY at pH 7.0, which was conducive to maintaining the stability of the conformation of secondary structures [42].

### 3.4. Particle Size Variation and Encapsulation Rate of EGCG by LY/Ps NPs

**Figure 6:**
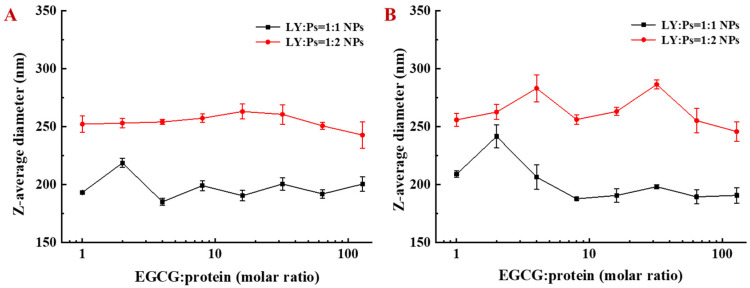
Particle size changes of lysozyme-pectin nanoparticles after (**A**) 0 h and (**B**) 24 h storage at room temperature.

The particle size of LY/Ps NPs, added with different EGCG concentrations, was measured using dynamic light scattering (DLS) for evaluating the stability of the NPs. At 0 h post-NPs’ storage time, we found that, at the mass ratio of LY to Ps of 1:1, different concentrations of EGCG addition into PBS solution (pH 7.0) did not change the particle size of LY/Ps NPs, whereas, at the mass ratio of LY to Ps of 1:2, the particle size of LY/Ps NPs was larger than that at the mass ratio of LY to Ps of 1:1 (Figure 6A). At 24 h post-NPs’ storage time, we observed that, when the molar ratio of EGCG to LY ranged from 1:1 to 128:1, the particle size of EGCG-LY/Ps NPs slightly changed, and when the mass ratio of LY to Ps was 1:1, the particle size was smaller than that when the mass ratio of LY to Ps was 1:2 (at the same concentration of EGCG) (Figure 6B). The particle size of the NPs placed at room temperature for 24 h exhibited no significant difference from that for 0 h, which indicated that EGCG might react with LY immediately after the addition of EGCG to LY solution.

When the molar ratio of EGCG to pure LY solution reached 8:1, precipitation occurred, which might be due to the direct bridging effect between EGCG and LY [37]. The binding models of EGCG to LY alone and LY/Ps NPs were different, which might be explained by the possibility that different phenolic groups of EGCG might bind different sites of LY. Overall, for the interaction of EGCG with the LY in LY/Ps NPs, the increase of EGCG concentration did not affect the particle size, and the EGCG-LY/Ps NPs solution was clear and transparent. The formation of insoluble particles in food beverages was undesirable and it also reduced the bioavailability of polyphenols, thus, our LY/Ps NPs were more suitable for application in food systems than pure protein solutions.

As can be seen from Table 2, the encapsulation rate of EGCG by LY/Ps NPs gradually decreased, but the loading of EGCG was gradually increased with the increasing molar ratio of EGCG to LY. Since polysaccharide attachment-induced steric hindrance hindered the formation of large particles, the particle size of LY/Ps NPs remained unchanged in spite of the increase of the molar ratio of EGCG to protein. Even when the molar ratio of EGCG to LY reached up to 128:1, the entrapment efficiency was still about 30%, and the content of EGCG in LY/Ps NPs was at a high level; therefore, LY/Ps NPs at different EGCG-LY molar ratios could be selected as the carrier of EGCG.

### 3.5. EGCG-LY/Ps NPs Enhanced the Resistance of C. Elegans to Heat Stress and Oxidative Stress

**Figure 7:**
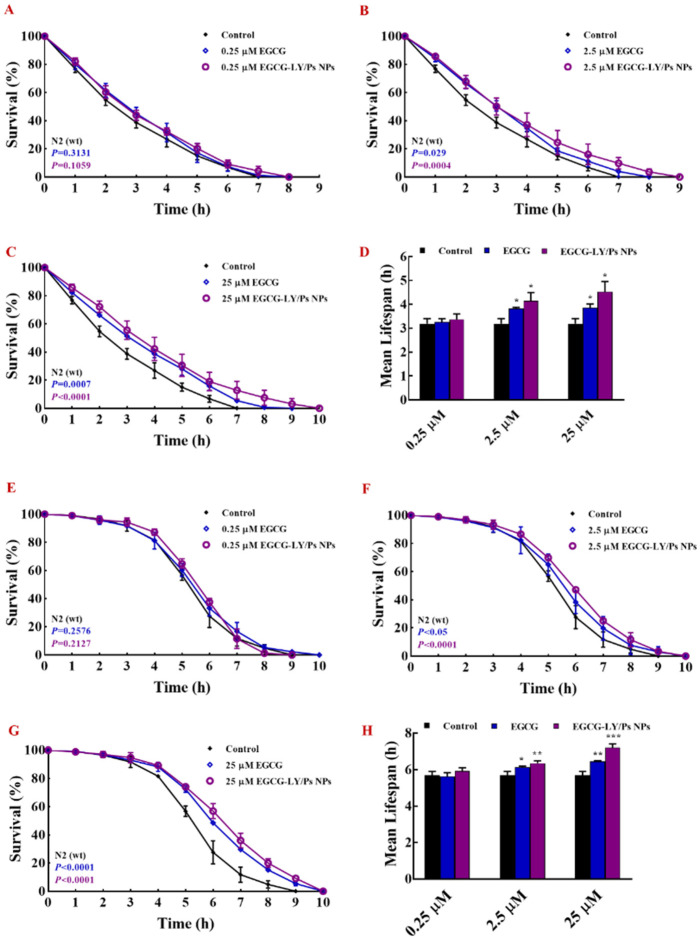
(**A**–**C**) Survival rates and (**D**) mean lifespan of *C. elegans* under heat stress. (**E**–**G**) Survival rates and (**H**) mean lifespan of *C. elegans* under oxidative stress under different concentrations of free EGCG and EGCG-lysozyme/pectin nanoparticles treatments.

Longevity of *C. elegans* is highly correlated with heat tolerance and oxidative stress [43,44]. The temperature of 15–25 °C is most suitable for the survival of nematodes, but when the temperature reaches 35 °C, the survival of nematodes will be greatly adversely affected, with denaturation and inactivation of enzymes in the organism [45]. The aging organisms produce large amounts of reactive oxygen species and superoxide. These oxides are accumulated in the organism, leading to oxidative damage [46]. Oxidative damage is closely associated with a variety of diseases, aging, and functional impairment. According to the previous experimental results, we chose the mass ratio of LY:Ps as 1:1 and the molar ratio of EGCG:LY as 1:1 to construct EGCG-LY/Ps nanoparticles. Under this condition, the entrapment efficiency of EGCG was 71.77 ± 8.01%. In this study, we explored the effects of free EGCG or EGCG-LY/Ps on the ability of *C. elegans* to resist heat stress and oxidative stress at three concentrations of 0.25 μM, 2.5 μM, and 25 μM. The nematodes were pretreated with EGCG-LY/Ps NPs or free EGCG for 5 days. The effects of different concentrations of EGCG treatments under the heat and oxidative stress conditions on the survival rate and life span of *C. elegans* were analyzed. The resulted showed that, at a low concentration (0.25 μM), EGCG had no effect on the survival rate of *C. elegans* under heat stress and paraquat oxidative stress, but with the increase in EGCG concentration, both free EGCG and EGCG in LY/Ps NPs significantly improved the survival rate of *C. elegans* under both stress conditions (Figure 7).

At medium concentration of EGCG (2.5 μM), the survival rate and average lifespan of nematodes in the EGCG-LY/Ps NPs (2.5 μM) group were significantly higher those in the free EGCG group (2.5 μM) under heat stress. At a high concentration of EGCG (25 μM), the survival rate and average life expectancy of nematodes in both groups were only slightly increased, compared with 2.5 μM EGCG treatment, but those in the EGCG-LY/Ps NPs (25 μM) group were still significantly higher than those of the free EGCG (25 μM) group under heat stress (Figure 7A–D). Under oxidative stress conditions, the survival rate and average lifespan of nematodes of both the medium (2.5 μM) and high concentration (25 μM) treatment groups were significantly increased, and at these two concentration levels, encapsulated EGCG exhibited a higher capability of enhancing nematodes’ resistance to oxidative stress than free EGCG. In addition, the resistance enhancement effect to oxidative stress become more pronounced with the increased concentration of encapsulated EGCG (Figure 7E–H). At 25 μM EGCG concentration, the mean lifespan of the free EGCG group was 113%, as high as that of the control group, while that of the EGCG-LY/Ps NPs group was 127%, as high as that of the control group; therefore, that of the EGCG-LY/Ps NPs group was 14% higher than that of the free EGCG group. Our results are in line with the previous report, showing that some polyphenols, such as blueberry polyphenols, tea polyphenols, and prunetin, can enhance the nematodes’ resistance to the stress, which is reflected by the prolonged life span of *C. elegans* under heat stress and oxidative stress conditions [47,48]. Likewise, the heat- and oxidation-stress resistance of nematodes fed with chitosan-pectin nanocarriers and encapsulated with anthocyanins is significantly higher than those fed with free-state anthocyanin [49]. In in-vitro cellular assays, EGCG significantly reduces the damage of mesenchymal stem cells under heat stress and significantly improves the morphology and viability of the cells [50]. In in-vivo experiments, EGCG can reduce the damage of heat stress and improve the growth performance of broilers by increasing the activity of antioxidant enzymes, such as superoxide dismutase and catalase in broilers [51]. EGCG can increase the ability of the *C. elegans* to resist heat stress by upregulating the expression of antioxidant enzymes and heat shock protein-16.2 [52]. In summary, our results showed that EGCG encapsulated in LY/Ps NPs more effectively improved *C. elegans’* resistance to heat and oxidative stresses than free-state EGCG, indicating that LY/Ps NPs can effectively enhance the activity of EGCG.

## 4. Conclusions

Lysozyme/pectin nanoparticles were prepared by the one-step self-assembly method, which could be efficiently loaded with EGCG. Proteins and polysaccharides combined to form spherical-structure nanoparticles through electrostatic interaction. The quenching of LY in LY/Ps NPs by EGCG was a static quenching process. Meanwhile, synchronous fluorescence showed that a red shift of the fluorescence peaks of tyrosine residues and tryptophan residues was observed, indicating that the addition of EGCG increased the polarity of the microenvironment and decreased the hydrophobicity of the residues. Circular dichroism analysis showed that the increase of α-helix content led to more folding of the LY structure and the increased stability of the helix structure. The increase of EGCG concentration did not obviously affect the particle size of NPs, suggesting that the NPs were of good stability. In addition, the LY/Ps NPs loaded with EGCG can prevent the precipitation of the EGCG-protein complex, which was conducive to a better entrapment effect. In vivo experiments showed that the stress resistance of *C. elegans* was significantly increased after incubation with EGCG-LY/Ps NPs and that the average lifespan increased by 142% and 127% under heat stress and oxidative stress, respectively. At the same concentration, the EGCG encapsulated in LY/Ps NPs exhibited a significantly better effect on the extension of *C. elegans*’ lifespan and the enhancement of their stress tolerance than free EGCG.

## Figures and Tables

**Table 1:** Binding parameters of EGCG-lysozyme, EGCG-nanoparticles, and thermodynamic parameters of the binding procedure.

Sample	T (K)	K_a_ (L·mol^−1^)	n	R^2^	ΔG (kJ·mol^−1^)	ΔS (J·mol^−1^·K^−1^)	ΔH (kJ·mol^−1^)
Native Protein	298	1.12 × 10^6^	1.4121	0.9995	−34.52	−324.57	−131.24
	308	2.01 × 10^5^	1.2217	0.9964	−31.27	−324.57	
LY:Ps = 1:1	298	9.01 × 10^4^	1.1677	0.9955	−28.26	−62.55	−46.90
	308	4.87 × 10^4^	1.1001	0.9976	−27.64	−62.53	
LY:Ps = 1:2	298	2.64 × 10^5^	1.2740	0.9937	−30.93	−4.73	−32.35
	308	1.73 × 10^5^	1.2170	0.9936	−30.88	−4.74	

**Table 2:** Effect of EGCG/lysozyme molar ratio (1:1) on EGCG loading onto EGCG-nanoparticles and entrapment efficiency of EGCG.

Molar Ratio (EGCG: Protein)	EGCG-Nanoparticles	
	Entrapment Efficiency (%)	EGCG Loading (μg/mg)
0:1	ND	ND
1:1	71.77 ± 8.01	11.48 ± 1.28
2:1	65.18 ± 5.01	20.85 ± 1.60
4:1	57.00 ± 4.08	36.48 ± 2.61
8:1	51.59 ± 3.31	66.03 ± 4.23
16:1	47.90 ± 3.05	122.62 ± 7.80
32:1	37.48 ± 2.70	191.90 ± 13.82
64:1	31.95 ± 1.59	327.17 ± 16.28
128:1	28.98 ± 1.28	593.51 ± 26.21

## Data Availability

The data presented in this study are available on request from the corresponding author.

## Supplementary Materials

The following are available online at https://www.mdpi.com/article/10.3390/foods10051127/s1, Figure S1: Linear plot of F_0_/F versus [EGCG] and linear plot of log(F_0_ − F)/F versus log [EGCG] (A, B) at 25 °C and (C, D) at 35 °C, Table S1: Secondary structure fractions of EGCG-lysozyme/pectin nanoparticles at pH 7.0, Table S2: Effect of EGCG/lysozyme molar ratio on ζ-potential and PDI of lysozyme/pectin nanoparticles on after 0 h storage at room temperature, Table S3: Effect of EGCG/lysozyme molar ratio on ζ-potential and PDI of lysozyme/pectin nanoparticles after 24 h storage at room temperature.
