# Supplementary material for: Construction and Application of EGCG-Loaded Lysozyme/Pectin Nanoparticles for Enhancing the Resistance of Nematodes to Heat and Oxidation Stresses
Source: Foods. 2021 May 19;10(5):1127. doi: 10.3390/foods10051127 (PMC8161057; doi:10.3390/foods10051127)
Supplement: Supplementary file 1 [file foods-10-01127-s001.zip › foods-1149479-supplementary.pdf]

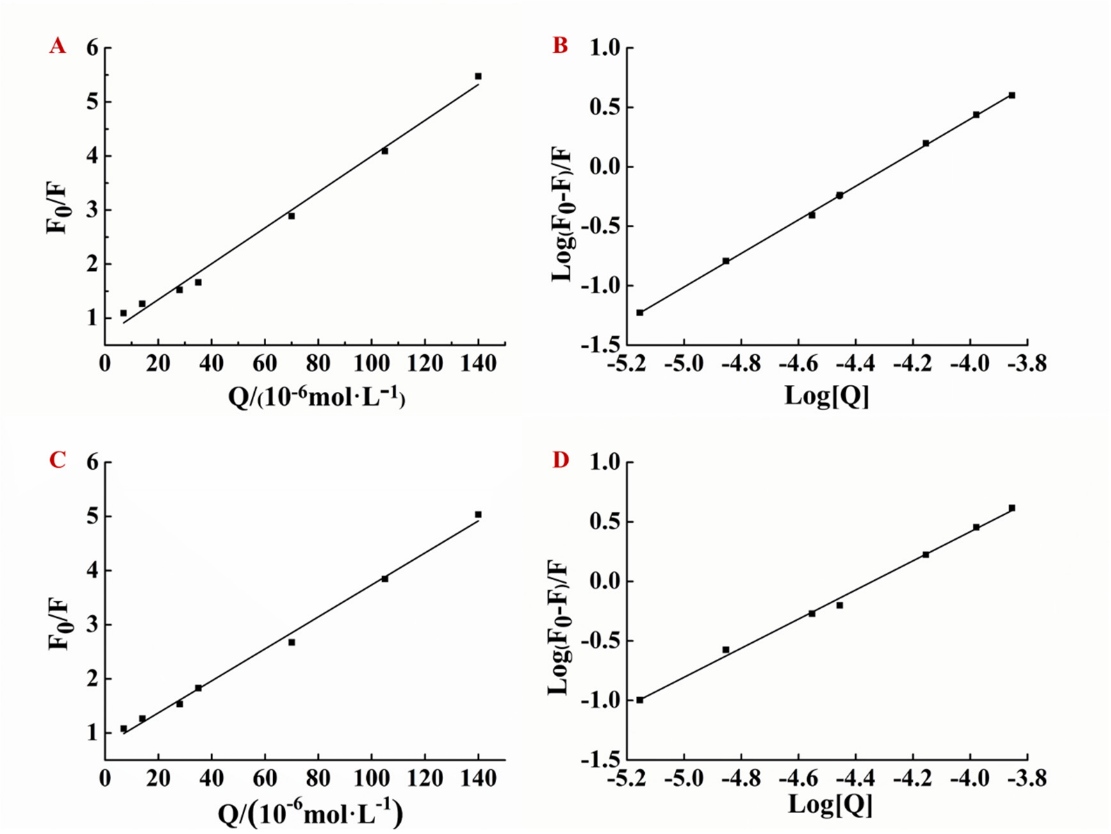

Figure S1. Linear plot of F_0/F versus [EGCG] and linear plot of $\log(F_0 - F)/F$ versus log [EGCG] (A, B) at 25 °C and (C, D) at 35 °C.

Table S1. Secondary structure fractions of EGCG-lysozyme/pectin nanoparticles at pH 7.0.

**EGCG/Protein Ratio**	**0:1**	**1:1**	**2:1**	**4:1**
α-helix (%)	21.0	23.4	29.3	34.5
β-sheet (%)	46.7	38.1	31.5	21.7
β-turn (%)	3.0	7.4	11.8	14.1
Random coil (%)	29.3	31.1	27.4	29.7

Table S2. Effect of EGCG/lysozyme molar ratio on ζ-potential and PDI of lysozyme/pectin nanoparticles on after 0 h storage at room temperature.

**Molar Ratio**	**LY: Ps = 1:1 NPs**	
**(EGCG: Protein)**	**ζ-Potential (mV)**	**PDI**
0:1	−34.8 ± 0.04	0.125 ± 0.01
1:1	−28.5 ± 1.42	0.254 ± 1.28
2:1	−36.3 ± 0.12	0.141 ± 0.01
4:1	−35.7 ± 0.02	0.138 ± 0.01
8:1	−35.5 ± 0.02	0.142 ± 0.01
16:1	−36.1 ± 0.02	0.178 ± 0.02
32:1	−35.8 ± 1.39	0.189 ± 1.56
64:1	−35.1 ± 0.11	0.135 ± 0.01
128:1	−34.3 ± 1.87	0.133 ± 2.12

Table S3. Effect of EGCG/lysozyme molar ratio on ζ-potential and PDI of lysozyme/pectin nanoparticles after 24 h storage at room temperature.

**Molar Ratio**	**LY: Ps = 1:1 NPs**	
**(EGCG: Protein)**	**ζ-Potential (mV)**	**PDI**
0:1	−32.8 ± 0.02	0.141 ± 0.02
1:1	−24.5 ± 1.51	0.377 ± 1.51
2:1	−37.8 ± 0.04	0.153 ± 0.01
4:1	−36.2 ± 0.02	0.156 ± 0.02
8:1	−38.5 ± 0.02	0.152 ± 0.01
16:1	−35.7 ± 0.04	0.190 ± 0.15
32:1	−35.8 ± 1.39	0.205 ± 1.48
64:1	−32.1 ± 0.25	0.153 ± 0.02
128:1	−35.9 ± 1.05	0.155 ± 2.51
